# Lärmbelästigung in der deutschen Allgemeinbevölkerung: Prävalenz und Determinanten in der Gutenberg-Gesundheitsstudie

**DOI:** 10.1007/s00059-021-05060-z

**Published:** 2021-08-13

**Authors:** Omar Hahad, Manfred Beutel, Matthias Michal, Andreas Schulz, Norbert Pfeiffer, Emilio Gianicolo, Karl Lackner, Philipp Wild, Andreas Daiber, Thomas Münzel

**Affiliations:** 1 Zentrum für Kardiologie – Kardiologie I, Universitätsmedizin der Johannes Gutenberg-Universität Mainz, Langenbeckstraße 1, 55131 Mainz, Deutschland; 2 Standort Rhein-Main, Deutsches Zentrum für Herz-Kreislauf-Forschung (DZHK), Mainz, Deutschland; 3 Klinik und Poliklinik für Psychosomatische Medizin und Psychotherapie, Universitätsmedizin der Johannes Gutenberg-Universität Mainz, Mainz, Deutschland; 4 Zentrum für Kardiologie – Präventive Kardiologie und Medizinische Prävention, Universitätsmedizin der Johannes Gutenberg-Universität Mainz, Mainz, Deutschland; 5 Augenklinik und Poliklinik, Universitätsmedizin der Johannes Gutenberg-Universität Mainz, Mainz, Deutschland; 6 Institut für Medizinische Biometrie, Epidemiologie und Informatik (IMBEI), Universitätsmedizin der Johannes Gutenberg-Universität Mainz, Mainz, Deutschland; 7 Institut für Klinische Chemie und Laboratoriumsmedizin, Universitätsmedizin der Johannes Gutenberg-Universität Mainz, Mainz, Deutschland

**Keywords:** Lärm, Gesundheitliche Einschränkungen, Soziodemographische Variablen, Kardiovaskuläre Risikofaktoren, Kohortenstudie, Noise, Impaired health, Sociodemographic variables, Cardiovascular risk factors, Cohort study

## Abstract

**Hintergrund:**

Lärmbelästigung, insbesondere durch Verkehrslärm, stellt ein massives Problem in der Bevölkerung dar und ist mit gesundheitlichen Einschränkungen assoziiert.

**Ziel der Arbeit:**

Anhand von Daten der bevölkerungsrepräsentativen Gutenberg-Gesundheitsstudie (GHS) werden die Prävalenz der Lärmbelästigung durch verschiedene Quellen sowie relevante Determinanten bestimmt.

**Material und Methoden:**

Die GHS ist eine populationsbasierte, prospektive Kohortenstudie in Deutschland, die Personen im Alter von 35 bis 74 Jahren einbezieht. 15.010 Probanden aus der Stadt Mainz und dem Landkreis Mainz-Bingen wurden von 2007 bis 2012 befragt, inwiefern sie sich in letzter Zeit durch Flug‑, Straßen‑, Schienen‑, Industrie- und Nachbarschaftslärm belästigt gefühlt haben (Angaben von „überhaupt nicht“ bis „äußerst“). Es wurde jeweils zwischen der Lärmbelästigung am Tag sowie während des Schlafens differenziert. Um die Beziehungen zwischen soziodemographischen Variablen, kardiovaskulären Risikofaktoren sowie Erkrankungen und Lärmbelästigung zu untersuchen, wurden multivariable logistische Regressionsmodelle verwendet.

**Ergebnisse:**

Etwa 80 % der Probanden fühlten sich durch Lärm belästigt. Fluglärmbelästigung am Tag stellte die vorherrschende Lärmbelästigungsquelle mit der höchsten Prävalenz stark (9,6 %) und äußerst lärmbelästigter Probanden dar (5,4 %), gefolgt von Straßenverkehrs- (stark: 4,0 %; äußerst: 1,6 %) und Nachbarschaftslärmbelästigung (stark: 3,5 %; äußerst: 1,3 %). Die Lärmbelästigung nahm eher mit zunehmender Altersdekade ab. Relevante Determinanten der Lärmbelästigung umfassten mitunter Geschlecht, Alter, sozioökonomischen Status, Depression, Angststörung, Schlafstörung und Vorhofflimmern.

**Diskussion:**

Lärmbelästigung betrifft einen Großteil der Bevölkerung und ist assoziiert mit soziodemographischen Variablen und kardiovaskulären Risikofaktoren sowie Erkrankungen.

**Zusatzmaterial online:**

Zusätzliche Informationen sind in der Online-Version dieses Artikels (10.1007/s00059-021-05060-z) enthalten.

## Abstract (en)

**Background:**

Noise annoyance, especially from traffic noise, is a massive problem in the population and is associated with impaired health.

**Objective:**

Based on data from the population representative Gutenberg Health Study (GHS), the prevalence of noise annoyance from different sources and relevant determinants were identified.

**Material and methods:**

The GHS is a population-based, prospective cohort study in Germany that included subjects aged 35–74 years. In the study 15,010 participants from the city of Mainz and the district of Mainz-Bingen were asked from 2007 to 2012 to what extent they had recently felt annoyed by aircraft, road, rail, industrial and neighborhood noise (answers ranged from not at all to extremely). A distinction was made between noise annoyance during the day and during sleep. To examine the relationships between sociodemographic variables, cardiovascular risk factors as well as diseases and noise annoyance, multivariable logistic regression models were used.

**Results:**

Approximately 80% of the participants felt annoyed by noise. Aircraft noise annoyance during the day was the predominant source of noise annoyance with the highest prevalence of strongly (9.6%) and extremely annoyed participants (5.4%), followed by road traffic (strongly 4.0% and extremely 1.6%) and neighborhood noise annoyance (strongly 3.5% and extremely 1.3%). Noise annoyance tended to decrease with increasing age. Relevant determinants of noise annoyance included gender, age, socioeconomic status, depression, anxiety disorder, sleep disorder and atrial fibrillation.

**Conclusion:**

Noise annoyance is common in the population and is associated with sociodemographic variables, cardiovascular risk factors and diseases.

## Einleitung

Lärm ist allgegenwärtig. Bereits vor über 100 Jahren wies der Nobelpreisträger für Medizin Robert Koch auf die drohende Gefahr einer Lärmepidemie mit dem Zitat hin: „Eines Tages wird der Mensch den Lärm ebenso unerbittlich bekämpfen müssen wie die Pest und die Cholera“ [1]. Inzwischen ist tatsächlich ein erheblicher Teil der Bevölkerung mit den negativen Auswirkungen von Lärm konfrontiert. Die Weltgesundheitsorganisation (WHO) geht davon aus, dass allein in Westeuropa lärmbedingt jährlich bis zu 1,6 Mio. gesunde Lebensjahre verloren gehen [2]. Zudem ist einem Bericht der European Environment Agency (EEA) zu entnehmen, dass Verkehrslärm in Europa, der die wichtigste Quelle für Lärm und Lärmbelästigung darstellt, für Lärmbelästigung bei 53 Mio. Erwachsenen sorgt [3]. In diesem Zusammenhang ist Verkehrslärm in Europa jährlich für 1,7 Mio. zusätzliche Fälle von Bluthochdruck verantwortlich, resultierend in 80.000 zusätzlichen Hospitalisierungen sowie 18.000 vorzeitigen Todesfällen aufgrund von koronarer Herzkrankheit (KHK) und Schlaganfall.

Ergebnisse zahlreicher epidemiologischer Studien zeigen, dass Verkehrslärmexposition mit erhöhter kardiovaskulärer Morbidität und Mortalität assoziiert ist [4–6]. Das Lärmwirkungsmodell von Babisch dient als Grundlage für die Charakterisierung der negativen Auswirkungen von Lärm auf den Organismus und schreibt der subjektiven Lärmbelästigung, also der individuellen Perzeption des Lärms durch den Lärmexponierten, eine zentrale Rolle bei der Vermittlung von kardiovaskulären Erkrankungen im Rahmen der nichtauditorischen Lärmwirkung zu [7–10]. In diesem Sinne können zu hohe Lärmpegel (weit unter der Auslöseschwelle zur Lärmschwerhörigkeit) über längere Zeiträume zu Beeinträchtigungen von Schlaf, Kommunikation und alltäglichen Aktivitäten führen und darüber kognitive und emotionale Stressreaktionen auslösen, die in Verbindung mit Lärmbelästigung stehen. Der Organismus reagiert darauf mit neuroendokrinen Stressreaktionen, also einem vermehrten Ausstoß von Stresshormonen, die langfristig zur Ausbildung/Manifestierung von klassischen kardiovaskulären Risikofaktoren wie Erhöhungen von Blutdruck, Blutzucker, Cholesterin, Blutviskosität und Blutgerinnung führen können. Persistieren diese Reaktionen über längere Zeiträume, kann dies zur Ausbildung/Manifestierung von kardiovaskulären Erkrankungen wie arterieller Hypertonie, Herzinsuffizienz, KHK und Schlaganfall führen [11]. Dies wurde vor Kurzem eindrucksvoll mittels Positronenemissionstomographie-Computertomographie (PET-CT) belegt, indem eine Aktivierung der Amygdala, eines Teils des limbischen Systems, verantwortlich für die emotionale Verarbeitung von Reizen, als Folge von Verkehrslärm nachgewiesen wurde [12]. Eine Mediationsanalyse der Ergebnisse durch die Autoren ergab, dass die durch Flug- und Straßenverkehrslärm ausgelöste Amygdalaaktivierung sekundär zu Entzündungen der Gefäße und nachfolgend zu vermehrten kardiovaskulären Ereignissen wie Herzinfarkt, Schlaganfall, koronare und periphere Revaskularisation sowie Tod führt. Exemplarisch wurde in einer Metaanalyse von Ndrepepa und Twardella unter Einschluss von 8 Studien gezeigt, dass Straßenverkehrslärmbelästigung mit einem erhöhten Risiko für Bluthochdruck und KHK assoziiert ist [13]. Hinsichtlich des Bluthochdrucks konnte eine gepoolte Risikoerhöhung (relatives Risiko [RR] und Odds Ratio [OR]) von 16 % 95 %-Konfidenzintervall [95 %-KI]: 1,02–1,29) beim Vergleich von keiner versus extreme bzw. niedrigere versus höhere Lärmbelästigung ermittelt werden, wobei bezüglich der KHK ein gepoolter Effektschätzer von 1,07 (95 %-KI: 0,99 –1,29) ermittelt wurde.

Daher stellt die Lärmbelästigung einen relevanten Gesundheitsindikator in der Bevölkerung dar. Das Ziel der vorliegenden Studie bestand darin, die Lärmbelästigung in der deutschen Allgemeinbevölkerung auf Basis einer großen populationsbasierten Kohorte, der Gutenberg-Gesundheitsstudie (GHS), zu charakterisieren und dabei relevante Determinanten der Lärmbelästigung zu identifizieren.

## Methoden

### Gutenberg-Gesundheitsstudie

Die GHS ist eine populationsbasierte, prospektive Kohortenstudie, die an der Universitätsmedizin Mainz durchgeführt wird [14]. Das in der GHS abgedeckte Studiengebiet umfasst die Stadt Mainz und den Landkreis Mainz-Bingen mit Einschluss von *n* = 15.010 Probanden (stratifiziert nach Altersdekaden, Geschlecht und Wohnort [Stadt vs. Land]) zur Baseline-Phase, die von 2007 bis 2012 durchgeführt wurde. Das Hauptanliegen der GHS besteht darin, bedeutsame Determinanten für Gesundheit und Krankheit in der Bevölkerung mit Fokus auf kardiovaskuläre, Augen‑, Krebs‑, Immun-, metabolische und psychische Erkrankungen zu identifizieren, um auf dieser Basis die Risikostratifizierung für den Einzelnen zu verbessern. Eingeschlossen wurden Personen im Alter von 35 bis 74 Jahren. Ausgeschlossen wurden Personen mit mangelnden Kenntnissen der deutschen Sprache sowie solche, die aufgrund von psychischen oder physischen Komplikationen nicht in der Lage waren, an den Untersuchungen im Studienzentrum teilzunehmen. Im Rahmen der hoch standardisierten 5‑stündigen Baseline-Untersuchung wurden die Probanden in das Studienzentrum eingeladen und einer umfassenden Testbatterie in einer definierten Reihenfolge unterzogen. Diese bestand aus einem computergestützten persönlichen Interview, umfangreichen medizinisch-technischen Untersuchungen, Befragungen mittels validierter Fragebögen und Biobanking und diente der Ermittlung von Faktoren, die den Lebensstil, Umwelteinflüsse, biopsychosoziale Variablen, laborchemische Parameter und die genetische Ausstattung umfassen. Alle in der GHS eingesetzten Untersuchungen unterliegen Standard Operating Procedures (SOP) und klar definierten Arbeitseinweisungen. Die GHS und ihre Studieninhalte wurden durch die Ethikkommission der Landesärztekammer Rheinland-Pfalz (Referenznummer: 837.020.07[5555]) und durch den Datenschutzbeauftragten der Universitätsmedizin Mainz geprüft und bewilligt. Die Studienprozeduren und -konzeption erfolgten unter Einhaltung des Bundesdatenschutzgesetzes und nach den Prinzipien der Deklaration von Helsinki, der Guten Klinischen Praxis (GCP) und der Guten Epidemiologischen Praxis (GEP) für die Planung, Durchführung, Dokumentation und Berichterstattung von klinischen/epidemiologischen Studien. Zu Studienbeginn wurde von allen Probanden eine informierte, schriftliche Einwilligungserklärung eingeholt.

### Lärmbelästigung

Die Erfassung der Lärmbelästigung erfolgte mittels einer international gebräuchlichen 5‑stufigen Likert-Skala, angelehnt an Felscher-Suhr, Guski und Schluemer [15]. Dabei wurden die Probanden gebeten, jeweils anzugeben (Kategorien von „überhaupt nicht“ bis „äußerst“), inwiefern sie sich in letzter Zeit durch Flug‑, Straßen‑, Schienen‑, Industrie- und Nachbarschaftslärm belästigt gefühlt haben. Dabei wurde jeweils nach der Lärmbelästigung am Tag sowie während des Schlafens gefragt. Tabelle e1 (E-Supplement) zeigt das Format dieser Angaben im Wortlaut.

### Bestimmung von soziodemographischen Variablen, kardiovaskulären Risikofaktoren und Erkrankungen

Im Rahmen der GHS wurde, wie oben beschrieben, eine umfangreiche und standardisierte Erfassung von soziodemographischen Variablen, kardiovaskulären Risikofaktoren und Erkrankungen, weiteren Surrogatmarkern, Lebensstilfaktoren sowie laborchemischen Parametern durchgeführt. Eine genaue Beschreibung der verwendeten Variablen in der vorliegenden Studie ist in Tabelle e2 (E-Supplement) enthalten.

### Statistische Analyse

Für die hier durchgeführten Analysen wurden die Daten der Baseline-Untersuchung herangezogen. Die Beschreibung der Probandencharakteristika erfolgte stratifiziert nach der totalen Lärmbelästigung. Angelehnt an Beutel et al. und Hahad et al. [16, 17] wurde die totale Lärmbelästigung definiert als maximal angegebene Belästigung, unabhängig von der spezifischen Lärmquelle (Flug‑, Straßen‑, Schienen‑, Industrie- oder Nachbarschaftslärm) und der Zeitperiode (am Tag oder beim Schlafen). Kategoriale Variablen wurden als absolute und/oder relative Häufigkeiten angegeben und kontinuierliche Variablen als Mittelwerte und Standardabweichungen. Um die Determinanten der Lärmbelästigung zu ermitteln, wurden multivariable logistische Regressionsmodelle mit Angabe von OR und 95 %-KI verwendet. Da die Kategorien „wenig“ bis „äußerst“ relativ gering besetzt waren, wurden im Rahmen der logistischen Regressionsanalysen die Kategorien „wenig“ bis „äußerst“ („überhaupt nicht“ vs. „wenig, mittel, stark und äußerst“) und die Kategorien „stark“ und „äußerst“ („überhaupt nicht, wenig und mittel“ vs. „stark und äußerst“) zusammengefasst. Jeweils separate Analysen wurden für die einzelnen Lärmbelästigungsquellen am Tag und beim Schlafen durchgeführt. Folgende Variablen wurden kategorial in die Regressionsmodelle aufgenommen:

- Geschlecht,
- Nachtschichtarbeit,
- Diabetes mellitus,
- Bluthochdruck,
- Rauchen,
- Adipositas,
- Dyslipidämie,
- Familiengeschichte von Herzinfarkt oder Schlaganfall,
- Alkoholkonsum oberhalb des tolerablen Grenzwerts,
- Depression,
- Angststörung,
- Schlafstörung,
- KHK,
- periphere arterielle Verschlusskrankheit (pAVK),
- Herzinfarkt,
- Herzinsuffizienz,
- Schlaganfall,
- Vorhofflimmern.

Folgende Variablen wurden kontinuierlich in die Regressionsmodelle aufgenommen:

- Alter,
- sozioökonomischer Status,
- Dauer des aktuellen Wohnsitzes,
- körperliche Aktivität.

Die statistischen Analysen wurden mit der Software R (Version 3.6.0, R Core Team [2019], Wien, Österreich) durchgeführt.

## Ergebnisse

### Probandencharakteristika

Zur Baseline-Phase der GHS machten insgesamt 14.639 Probanden Angaben zur Lärmbelästigung (Tab. 1). In Bezug auf die totale Lärmbelästigung fühlten sich 20,7 % der Probanden überhaupt nicht durch Lärm belästigt, wobei sich 26,6 % wenig, 25,0 % mittelgradig, 17,3 % stark und 10,5 % äußerst belästigt fühlten. Die Gruppe äußerst lärmbelästigter Probanden besaß die höchste Prävalenz weiblichen Geschlechts (54,4 %), gefolgt von der überhaupt nicht lärmbelästigten Gruppe (51,5 %). Die wenig lärmbelästigten Probanden waren die jüngsten (53,8 Jahre), am ältesten waren dagegen die überhaupt nicht lärmbelästigten Probanden (56,3 Jahre). Überhaupt nicht lärmbelästige Probanden besaßen den geringsten sozioökonomischen Status (12,18) und wenig lärmbelästige Probanden den höchsten sozioökonomischen Status (13,47). Starke (24,1 %) und äußerste (23,8 %) Lärmbelästigung war mit der höchsten Prävalenz von Nachtschichtarbeit verbunden. Bezüglich der Verteilung von kardiovaskulären Risikofaktoren ergab sich ein eher schlechteres Risikoprofil für die überhaupt nicht lärmbelästigten Probanden. Diese waren körperlich weniger aktiv (7,26) und hatten die höchste Prävalenz von Diabetes mellitus (11,0 %), Bluthochdruck (51,9 %), Rauchen (21,8 %), Adipositas (28,4 %) und einer positiven Familiengeschichte von Herzinfarkt oder Schlaganfall (23,7 %). Dagegen war eine stärkere Lärmbelästigung eher mit einer höheren Prävalenz von Depressionen, Angststörungen, Schlafstörungen und übermäßigem Alkoholkonsum verbunden. Hinsichtlich der Prävalenz von kardiovaskulären Erkrankungen ergab sich dagegen keine klare Verteilung in Bezug auf die totale Lärmbelästigung. Probanden mit äußerster Lärmbelästigung hatten die höchste Prävalenz von pAVK (4,0 %) und Vorhofflimmern (23,6 %). Dagegen war die höchste Prävalenz von Schlaganfall (2,2 %) und Herzinfarkt (3,6 %) bei Probanden, die sich überhaupt nicht lärmbelästigt fühlten, zu beobachten.

**Table 1:** 

Charakteristika	Totale Lärmbelästigung				
	Überhaupt nicht (*n* = 3024, 20,7 %)	Wenig (*n* = 3895, 26,6 %)	Mittel (*n* = 3654, 25,0 %)	Stark (*n* = 2536, 17,3 %)	Äußerst (*n* = 1530, 10,5 %)
*Soziodemographische Variablen*					
Weibliches Geschlecht (%)	51,5	45,6	49,6	49,5	54,4
Alter in Jahren	56,3 ± 11,0	53,8 ± 11,0	55,1 ± 11,3	54,5 ± 11,2	54,9 ± 10,7
Sozioökonomischer Status	12,18 ± 4,44	13,47 ± 4,42	12,91 ± 4,44	13,04 ± 4,52	12,84 ± 4,41
Dauer des aktuellen Wohnsitzes in Jahren	20,35 ± 15,41	18,20 ± 14,32	20,02 ± 15,38	19,06 ± 14,76	18,66 ± 14,69
Nachtschichtarbeit (%)	21,5	23,1	21,9	24,1	23,8
*Kardiovaskuläre Risikofaktoren*					
Diabetes mellitus (%)	11,0	7,7	9,1	8,9	8,6
Bluthochdruck (%)	51,9	47,4	50,7	48,8	47,3
Rauchen (%)	21,8	19,2	18,6	17,6	21,2
Adipositas (%)	28,4	23,0	24,2	25,6	24,0
Dyslipidämie (%)	45,2	42,5	44,0	43,8	45,7
Familiengeschichte von Herzinfarkt oder Schlaganfall (%)	23,7	20,2	22,1	22,0	22,5
Alkoholkonsum oberhalb des tolerablen Grenzwerts (%)	22,2	22,0	23,1	22,5	23,3
Depression (%)	6,1	5,8	7,2	9,6	12,0
Angststörung (%)	4,5	5,4	6,5	8,0	10,0
Schlafstörung (%)	16,7	16,4	20,2	21,9	26,5
Körperliche Aktivität	7,26 ± 4,27	7,51 ± 3,82	7,34 ± 3,86	7,34 ± 3,99	7,52 ± 4,05
*Kardiovaskuläre Erkrankungen*					
Koronare Herzkrankheit (%)	4,1	3,7	4,4	4,3	4,4
Periphere arterielle Verschlusskrankheit (%)	3,6	2,6	3,2	3,5	4,0
Herzinfarkt (%)	3,6	2,5	2,8	2,6	3,1
Herzinsuffizienz (%)	7,9	7,3	7,9	7,3	7,1
Schlaganfall (%)	2,2	1,5	1,9	1,5	1,2
Vorhofflimmern (%)	14,6	16,0	18,0	20,1	23,6

Dargestellt sind Mittelwerte ± Standardabweichungen oder relative Häufigkeiten in Prozent

### Prävalenz von Lärmbelästigung

**Figure 1:**
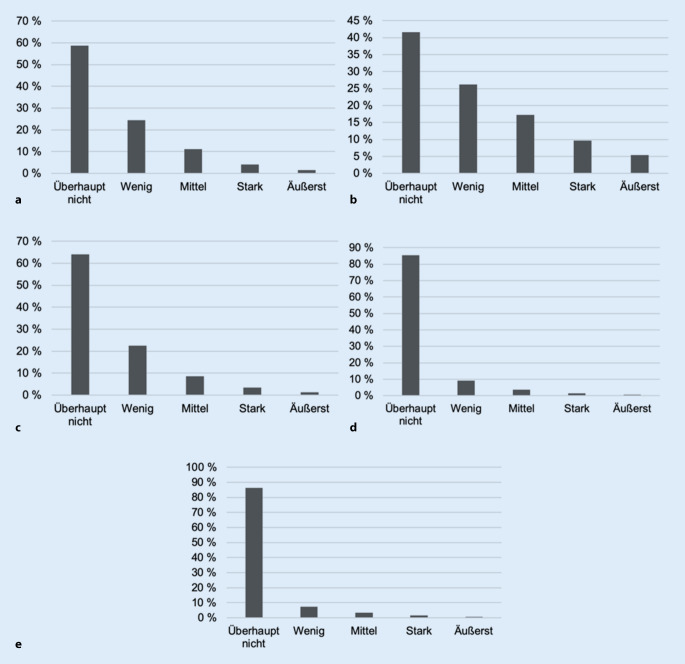

**Figure 2:**
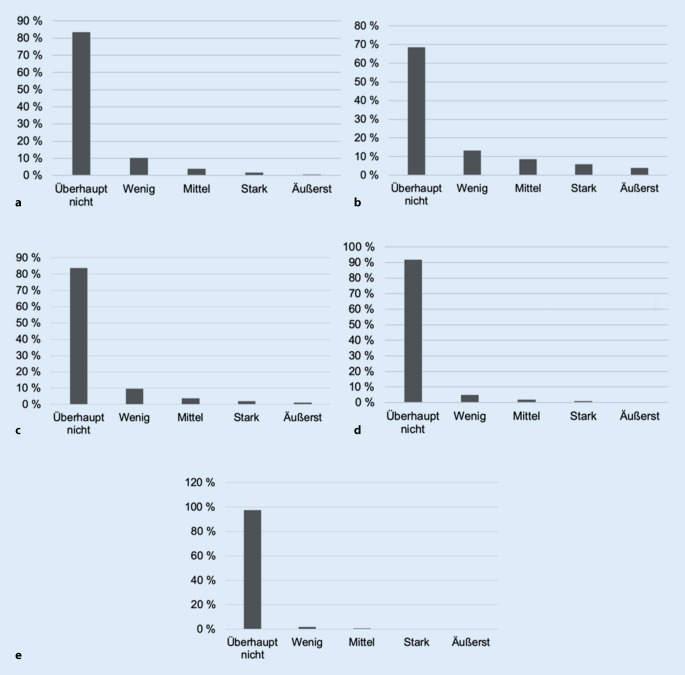

Wie in Abb. 1 ersichtlich, stellte die Fluglärmbelästigung am Tag die vorherrschende Lärmbelästigungsquelle mit der höchsten Prävalenz stark (9,6 %) und äußerst lärmbelästigter Probanden dar (5,4 %), gefolgt von der Straßenverkehrs- (stark: 4,0 % und äußerst: 1,6 %) und Nachbarschaftslärmbelästigung (stark: 3,5 % und äußerst: 1,3 %). Hinsichtlich der Lärmbelästigung beim Schlafen (Abb. 2) zeigte sich eine ähnliche Verteilung, jedoch war die Lärmbelästigung beim Schlafen insgesamt weniger stark ausgeprägt als am Tag.

### Lärmbelästigung, Alter und Geschlecht

Tab. 2 zeigt die Prävalenz lärmbelästigter Probanden (> 0), stratifiziert nach Altersdekaden (35–44, 45–54, 55–64 und 65–74 Jahre) und Geschlecht, an. In der Gesamtpopulation nahm die Prävalenz lärmbelästiger Probanden (totale Lärmbelästigung) über die zunehmenden Altersdekaden ab (35–44: 82,7 %, 45–44: 81,1 %, 55–64: 78,2 % und 65–74: 75,6 %). Dies konnte ebenfalls für die spezifischen Lärmbelästigungsquellen beobachtet werden, ausgenommen für die Fluglärmbelästigung, die am Tag (35–44: 55,8 %; 45–44: 59,7 %; 55–64: 59,6 % und 65–74: 58,0 %) und beim Schlafen (35–44: 29,4 %; 45–44: 33,4 %; 55–64: 32,7 % und 65–74: 30,1 %) eher zunahm. Diese Verteilung wurde auch bei separater Betrachtung von Männern und Frauen beobachtet.

**Table 2:** 

Lärmbelästigung	Altersdekaden			
	35–44	45–54	55–64	65–74
**Gesamtpopulation**				
*Am Tag (>* *0)*				
Straßenverkehrslärm	40,8 (1328)	42,6 (1672)	41,4 (1595)	40,0 (1440)
Fluglärm	55,8 (1819)	59,7 (2339)	59,6 (2299)	58,0 (2087)
Schienenverkehrslärm	16,0 (521)	15,9 (622)	13,4 (514)	13,0 (466)
Industrie‑, Bau- und Gewerbelärm	18,8 (611)	16,1 (630)	11,2 (431)	8,2 (295)
Nachbarschaftslärm	49,3 (1605)	41,2 (1616)	30,4 (1173)	24,1 (869)
*Beim Schlafen (>* *0)*				
Straßenverkehrslärm	18,3 (594)	18,1 (710)	15,9 (612)	13,2 (474)
Fluglärm	29,4 (956)	33,4 (1307)	32,7 (1257)	30,1 (1079)
Schienenverkehrslärm	8,7 (284)	9,5 (371)	7,8 (298)	6,3 (225)
Industrie‑, Bau- und Gewerbelärm	3,8 (123)	3,0 (116)	2,5 (96)	1,3 (46)
Nachbarschaftslärm	22,3 (725)	19,8 (774)	13,7 (526)	9,7 (348)
*Totale Lärmbelästigung (>* *0)*	82,7 (2694)	81,1 (3182)	78,2 (3016)	75,6 (2723)
**Männer**				
*Am Tag (>* *0)*				
Straßenverkehrslärm	41,3 (651)	43,4 (859)	42,1 (828)	42,2 (794)
Fluglärm	56,9 (897)	60,8 (1203)	62,5 (1228)	61,9 (1164)
Schienenverkehrslärm	16,6 (261)	16,8 (332)	14,1 (277)	14,8 (278)
Industrie‑, Bau- und Gewerbelärm	20,7 (325)	16,4 (324)	11,8 (232)	9,3 (174)
Nachbarschaftslärm	49,3 (776)	40,4 (799)	31,2 (614)	26,3 (495)
*Beim Schlafen (>* *0)*				
Straßenverkehrslärm	17,3 (273)	18,4 (363)	15,8 (310)	13,5 (252)
Fluglärm	28,5 (448)	33,6 (663)	34,8 (684)	33,9 (634)
Schienenverkehrslärm	9,0 (141)	10,3 (204)	8,0 (158)	7,4 (139)
Industrie‑, Bau- und Gewerbelärm	4,6 (72)	3,1 (61)	3,1 (61)	1,7 (31)
Nachbarschaftslärm	21,8 (343)	18,9 (373)	12,9 (254)	9,0 (169)
*Totale Lärmbelästigung (>* *0)*	81,9 (1291)	81,3 (1612)	79,2 (1559)	78,6 (1477)
**Frauen**				
*Am Tag (>* *0)*				
Straßenverkehrslärm	40,3 (677)	41,9 (813)	40,6 (767)	37,5 (646)
Fluglärm	54,8 (922)	58,6 (1136)	56,7 (1071)	53,6 (923)
Schienenverkehrslärm	15,5 (260)	15,0 (290)	12,6 (237)	10,9 (188)
Industrie‑, Bau- und Gewerbelärm	17,0 (286)	15,8 (306)	10,6 (199)	7,0 (121)
Nachbarschaftslärm	49,3 (829)	42,1 (817)	29,6 (559)	21,7 (374)
*Beim Schlafen (>* *0)*				
Straßenverkehrslärm	19,2 (321)	17,9 (347)	16,0 (302)	12,9 (222)
Fluglärm	30,3 (508)	33,2 (644)	30,4 (573)	26,0 (445)
Schienenverkehrslärm	8,5 (143)	8,6 (167)	7,4 (140)	5,0 (86)
Industrie‑, Bau- und Gewerbelärm	3,0 (51)	2,8 (55)	1,9 (35)	0,9 (15)
Nachbarschaftslärm	22,8 (382)	20,7 (401)	14,5 (272)	10,4 (179)
*Totale Lärmbelästigung (>* *0)*	83,5 (1403)	80,9 (1570)	77,1 (1457)	72,4 (1246)

Dargestellt als relative und absolute Häufigkeiten

### Lärmbelästigung, Geschlecht und kardiovaskuläre Erkrankungen

79,3 % aller Probanden gaben an, von Lärmbelästigung betroffen zu sein (totale Lärmbelästigung > 0; (Tabelle e3 [E-Supplement]). Vorherrschende Lärmbelästigungsquellen (> 0) in der Gesamtpopulation am Tag waren Fluglärm (58,4 %), Straßenverkehrslärm (41,2 %) und Nachbarschaftslärm (36,0 %). Diese Quellen waren auch in der Schlafphase vorherrschend (Fluglärm: 31,5 %, Straßenverkehrslärm: 16,4 % und Nachbarschaftslärm: 16,3 %). Fluglärm am Tag sorgte sowohl bei Männern (60,7 %) als auch bei Frauen (56,0 %) für die höchste Prävalenz lärmbelästigter Probanden. Männer (80,2 %) waren geringfügig häufiger von Lärmbelästigung betroffen als Frauen (78,5 %; totale Lärmbelästigung > 0). Die Prävalenz lärmbelästiger Probanden am Tag war in der Gesamtpopulation höher als beim Schlafen.

Tabelle e4 (E-Supplement) zeigt die Prävalenz der lärmbelästigten Probanden (> 0), stratifiziert nach kardiovaskulären Erkrankungen und Geschlecht, an. Hinsichtlich der totalen Lärmbelästigung wurde in der Gesamtpopulation bei Vorliegen von pAVK, Herzinfarkt, Herzinsuffizienz und Schlaganfall eine eher geringere Prävalenz von Lärmbelästigung beobachtet, wobei das Vorliegen einer KHK und von Vorhofflimmern mit einer höheren Prävalenz lärmbelästigter Probanden verbunden war.

### Determinanten der Lärmbelästigung

In Tab. 3 sind die Ergebnisse der logistischen Regressionsanalysen im Hinblick auf die Determinanten der Lärmbelästigung dargestellt. Die stärksten Determinanten der Lärmbelästigung umfassten mitunter Geschlecht, Alter, sozioökonomischen Status, Depression, Angststörung, Schlafstörung und Vorhofflimmern. Tendenziell fühlten sich Frauen und Probanden höheren Alters weniger häufig durch Lärm belästigt, wobei die Richtung des Zusammenhangs für den sozioökonomischen Status weniger konsistent zu bewerten war. Das Vorhandensein von Depression, Angststörung, Schlafstörung und von Vorhofflimmern war mit erhöhter Lärmbelästigung assoziiert.

**Table 3:** 

Determinanten	OR (95 %-KI) Wenig bis äußerst	OR (95 %-KI) Stark und äußerst
**Straßenverkehrslärm**		
*Am Tag*		
Weibliches Geschlecht	*0,88 (0,82–0,95)*	0,94 (0,80–1,09)
Alter	*0,95 (0,93–0,97)*	*0,94 (0,90–0,98)*
Sozioökonomischer Status	*0,97 (0,96–0,98)*	*0,94 (0,92–0,96)*
Dauer des aktuellen Wohnsitzes	*1,03 (1,01–1,04)*	1,03 (1,00–1,06)
Nachtschichtarbeit	*1,16 (1,07–1,26)*	1,11 (0,93–1,31)
Diabetes mellitus	0,91 (0,80–1,04)	1,00 (0,77–1,29)
Bluthochdruck	1,05 (0,97–1,14)	0,96 (0,81–1,13)
Rauchen	*0,91 (0,84–1,00)*	0,92 (0,76–1,11)
Adipositas	0,94 (0,86–1,02)	1,01 (0,85–1,20)
Dyslipidämie	1,05 (0,97–1,13)	1,11 (0,95–1,30)
Familiengeschichte von Herzinfarkt oder Schlaganfall	0,94 (0,87–1,02)	0,92 (0,77–1,09)
Alkoholkonsum oberhalb des tolerablen Grenzwerts	0,96 (0,89–1,05)	1,09 (0,91–1,29)
Depression	1,14 (0,98–1,33)	1,33 (0,99–1,76)
Angststörung	*1,27 (1,09–1,49)*	1,27 (0,94–1,69)
Schlafstörung	*1,16 (1,06–1,28)*	1,17 (0,96–1,41)
Körperliche Aktivität	0,99 (0,98–1,00)	0,99 (0,96–1,01)
Koronare Herzkrankheit	1,16 (0,95–1,43)	1,11 (0,73–1,64)
Periphere arterielle Verschlusskrankheit	0,95 (0,78–1,15)	1,16 (0,78–1,68)
Herzinfarkt	0,80 (0,62–1,02)	0,95 (0,56–1,55)
Herzinsuffizienz	1,04 (0,92–1,19)	0,97 (0,73–1,27)
Schlaganfall	*0,72 (0,55–0,95)*	0,64 (0,31–1,15)
Vorhofflimmern	1,09 (0,99–1,19)	*1,26 (1,05–1,50)*
*Beim Schlafen*		
Weibliches Geschlecht	*0,90 (0,82–1,00)*	1,11 (0,87–1,43)
Alter	*0,91 (0,88–0,94)*	*0,93 (0,86–0,99)*
Sozioökonomischer Status	*0,98 (0,97–0,99)*	*0,96 (0,93–0,99)*
Dauer des aktuellen Wohnsitzes	1,02 (1,00–1,04)	0,97 (0,92–1,02)
Nachtschichtarbeit	1,00 (0,89–1,11)	1,15 (0,87–1,49)
Diabetes mellitus	0,89 (0,74–1,06)	0,89 (0,56–1,35)
Bluthochdruck	0,97 (0,88–1,08)	0,95 (0,74–1,23)
Rauchen	*0,72 (0,64–0,81)*	0,87 (0,64–1,15)
Adipositas	0,93 (0,83–1,04)	1,36 (1,05–1,77)
Dyslipidämie	0,99 (0,90–1,09)	0,91 (0,71–1,17)
Familiengeschichte von Herzinfarkt oder Schlaganfall	0,95 (0,85–1,06)	1,02 (0,78–1,33)
Alkoholkonsum oberhalb des tolerablen Grenzwerts	1,03 (0,93–1,15)	0,94 (0,70–1,24)
Depression	1,13 (0,94–1,37)	1,14 (0,75–1,70)
Angststörung	*1,27 (1,05–1,53)*	*2,03 (1,38–2,94)*
Schlafstörung	*1,47 (1,31–1,66)*	*1,44 (1,08–1,90)*
Körperliche Aktivität	1,00 (0,98–1,01)	1,00 (0,96–1,03)
Koronare Herzkrankheit	1,11 (0,84–1,46)	0,54 (0,23–1,15)
Periphere arterielle Verschlusskrankheit	1,11 (0,85–1,43)	1,56 (0,87–2,60)
Herzinfarkt	0,83 (0,57–1,18)	1,87 (0,82–3,85)
Herzinsuffizienz	0,99 (0,82–1,17)	0,89 (0,55–1,37)
Schlaganfall	0,88 (0,59–1,28)	0,78 (0,24–1,88)
Vorhofflimmern	*1,22 (1,08–1,37)*	*1,58 (1,21–2,05)*
**Fluglärm**		
*Am Tag*		
Weibliches Geschlecht	*0,87 (0,81–0,93)*	1,02 (0,92–1,13)
Alter	*1,03 (1,01–1,05)*	*1,06 (1,03–1,09)*
Sozioökonomischer Status	*1,06 (1,06–1,07)*	*1,04 (1,03–1,05)*
Dauer des aktuellen Wohnsitzes	*1,03 (1,01–1,04)*	1,01 (0,99–1,02)
Nachtschichtarbeit	0,95 (0,87–1,03)	1,06 (0,94–1,18)
Diabetes mellitus	0,95 (0,84–1,08)	0,95 (0,79–1,13)
Bluthochdruck	0,98 (0,91–1,06)	0,93 (0,84–1,04)
Rauchen	*0,80 (0,73–0,87)*	*0,86 (0,76–0,97)*
Adipositas	*0,88 (0,81–0,96)*	*0,89 (0,79–1,00)*
Dyslipidämie	1,04 (0,97–1,12)	1,05 (0,95–1,16)
Familiengeschichte von Herzinfarkt oder Schlaganfall	0,95 (0,88–1,03)	1,01 (0,90–1,13)
Alkoholkonsum oberhalb des tolerablen Grenzwerts	1,04 (0,96–1,13)	1,00 (0,89–1,12)
Depression	1,03 (0,88–1,21)	1,16 (0,94–1,42)
Angststörung	1,09 (0,93–1,28)	1,14 (0,92–1,40)
Schlafstörung	1,08 (0,98–1,19)	1,15 (1,01–1,30)
Körperliche Aktivität	1,00 (0,99–1,01)	1,00 (0,98–1,01)
Koronare Herzkrankheit	1,12 (0,91–1,38)	1,12 (0,85–1,46)
Periphere arterielle Verschlusskrankheit	1,08 (0,89–1,32)	1,26 (0,98–1,62)
Herzinfarkt	*0,69 (0,53–0,88)*	0,93 (0,66–1,30)
Herzinsuffizienz	0,92 (0,81–1,05)	0,96 (0,80–1,15)
Schlaganfall	*0,67 (0,51–0,87)*	*0,65 (0,42–0,96)*
Vorhofflimmern	*1,13 (1,03–1,24)*	1,11 (0,98–1,25)
*Beim Schlafen*		
Weibliches Geschlecht	*0,91 (0,84–0,99)*	*1,15 (1,02–1,31)*
Alter	1,02 (1,00–1,04)	*1,07 (1,03–1,11)*
Sozioökonomischer Status	*1,07 (1,06–1,08)*	*1,08 (1,06–1,09)*
Dauer des aktuellen Wohnsitzes	*1,03 (1,01–1,04)*	1,01 (0,99–1,04)
Nachtschichtarbeit	0,98 (0,89–1,07)	1,04 (0,91–1,19)
Diabetes mellitus	0,94 (0,82–1,08)	1,00 (0,80–1,24)
Bluthochdruck	0,93 (0,86–1,01)	*0,83 (0,73–0,94)*
Rauchen	*0,75 (0,68–0,82)*	0,88 (0,75–1,02)
Adipositas	*0,82 (0,75–0,90)*	0,88 (0,76–1,02)
Dyslipidämie	1,01 (0,94–1,10)	1,03 (0,91–1,16)
Familiengeschichte von Herzinfarkt oder Schlaganfall	1,05 (0,96–1,15)	1,07 (0,94–1,23)
Alkoholkonsum oberhalb des tolerablen Grenzwerts	1,08 (0,99–1,17)	0,97 (0,85–1,11)
Depression	0,95 (0,80–1,13)	1,11 (0,87–1,41)
Angststörung	1,13 (0,96–1,34)	*1,29 (1,01–1,63)*
Schlafstörung	*1,26 (1,14–1,39)*	*1,22 (1,05–1,41)*
Körperliche Aktivität	1,00 (0,99–1,01)	1,01 (0,99–1,02)
Koronare Herzkrankheit	1,14 (0,92–1,42)	1,27 (0,92–1,73)
Periphere arterielle Verschlusskrankheit	0,96 (0,77–1,19)	1,19 (0,87–1,61)
Herzinfarkt	0,76 (0,57–1,01)	0,93 (0,61–1,39)
Herzinsuffizienz	1,02 (0,89–1,18)	1,04 (0,83–1,29)
Schlaganfall	0,78 (0,57–1,05)	*0,52 (0,28–0,88)*
Vorhofflimmern	*1,23 (1,12–1,35)*	*1,31 (1,14–1,51)*
**Schienenverkehrslärm**		
*Am Tag*		
Weibliches Geschlecht	*0,83 (0,75–0,92)*	0,89 (0,68–1,16)
Alter	0,97 (0,94–1,00)	*0,92 (0,86–1,00)*
Sozioökonomischer Status	0,99 (0,98–1,00)	*0,92 (0,90–0,95)*
Dauer des aktuellen Wohnsitzes	0,98 (0,96–1,00)	1,01 (0,96–1,06)
Nachtschichtarbeit	1,03 (0,91–1,15)	1,30 (0,97–1,71)
Diabetes mellitus	0,87 (0,72–1,04)	0,83 (0,51–1,29)
Bluthochdruck	0,92 (0,83–1,03)	0,95 (0,72–1,25)
Rauchen	0,96 (0,85–1,08)	1,01 (0,74–1,35)
Adipositas	1,04 (0,92–1,17)	1,01 (0,75–1,34)
Dyslipidämie	0,99 (0,89–1,09)	1,04 (0,80–1,36)
Familiengeschichte von Herzinfarkt oder Schlaganfall	1,05 (0,94–1,18)	0,85 (0,62–1,14)
Alkoholkonsum oberhalb des tolerablen Grenzwerts	1,11 (1,00–1,25)	0,95 (0,69–1,28)
Depression	*1,27 (1,03–1,56)*	*2,01 (1,26–3,15)*
Angststörung	1,14 (0,92–1,40)	1,40 (0,87–2,19)
Schlafstörung	0,98 (0,86–1,12)	0,80 (0,55–1,12)
Körperliche Aktivität	1,01 (0,99–1,02)	1,00 (0,97–1,03)
Koronare Herzkrankheit	1,04 (0,78–1,37)	1,1 (0,55–2,10)
Periphere arterielle Verschlusskrankheit	1,24 (0,95–1,60)	1,54 (0,84–2,62)
Herzinfarkt	1,23 (0,87–1,71)	2,04 (0,97–4,06)
Herzinsuffizienz	1,08 (0,90–1,28)	0,86 (0,52–1,35)
Schlaganfall	1,03 (0,70–1,48)	0,58 (0,14–1,56)
Vorhofflimmern	1,06 (0,94–1,20)	1,01 (0,73–1,38)
*Beim Schlafen*		
Weibliches Geschlecht	*0,79 (0,69–0,90)*	0,88 (0,64–1,21)
Alter	*0,95 (0,91–0,98)*	*0,90 (0,82–0,99)*
Sozioökonomischer Status	1,00 (0,98–1,01)	0,96 (0,93–1,00)
Dauer des aktuellen Wohnsitzes	*0,97 (0,95–1,00)*	1,02 (0,96–1,08)
Nachtschichtarbeit	1,00 (0,86–1,16)	1,32 (0,94–1,83)
Diabetes mellitus	0,87 (0,68–1,10)	0,97 (0,54–1,63)
Bluthochdruck	0,91 (0,79–1,04)	1,00 (0,72–1,39)
Rauchen	*0,70 (0,59–0,83)*	0,74 (0,49–1,08)
Adipositas	0,98 (0,84–1,14)	0,81 (0,56–1,16)
Dyslipidämie	1,02 (0,89–1,17)	1,04 (0,75–1,42)
Familiengeschichte von Herzinfarkt oder Schlaganfall	1,02 (0,88–1,18)	1,00 (0,70–1,40)
Alkoholkonsum oberhalb des tolerablen Grenzwerts	1,12 (0,97–1,30)	0,78 (0,53–1,14)
Depression	1,17 (0,91–1,51)	1,52 (0,86–2,60)
Angststörung	*1,34 (1,04–1,72)*	1,42 (0,81–2,40)
Schlafstörung	*1,19 (1,00–1,40)*	1,03 (0,69–1,52)
Körperliche Aktivität	0,99 (0,98–1,01)	0,98 (0,94–1,03)
Koronare Herzkrankheit	1,10 (0,76–1,56)	1,07 (0,44–2,29)
Periphere arterielle Verschlusskrankheit	1,24 (0,87–1,71)	1,34 (0,59–2,62)
Herzinfarkt	1,23 (0,79–1,88)	1,76 (0,67–4,16)
Herzinsuffizienz	1,09 (0,86–1,36)	0,75 (0,39–1,32)
Schlaganfall	1,25 (0,78–1,92)	0,59 (0,10–1,88)
Vorhofflimmern	*1,22 (1,04–1,42)*	*1,60 (1,13–2,24)*
**Industrie‑, Bau- und Gewerbelärm**		
*Am Tag*		
Weibliches Geschlecht	*0,86 (0,77–0,96)*	1,10 (0,87–1,39)
Alter	*0,85 (0,82–0,87)*	*0,87 (0,81–0,93)*
Sozioökonomischer Status	1,00 (0,99–1,01)	0,98 (0,95–1,00)
Dauer des aktuellen Wohnsitzes	0,99 (0,97–1,01)	0,99 (0,94–1,04)
Nachtschichtarbeit	*1,20 (1,06–1,34)*	1,07 (0,82–1,39)
Diabetes mellitus	0,91 (0,74–1,11)	0,81 (0,50–1,24)
Bluthochdruck	1,01 (0,90–1,13)	1,19 (0,93–1,51)
Rauchen	0,92 (0,81–1,04)	1,04 (0,79–1,34)
Adipositas	1,12 (0,99–1,26)	1,24 (0,96–1,60)
Dyslipidämie	1,02 (0,92–1,14)	1,01 (0,80–1,28)
Familiengeschichte von Herzinfarkt oder Schlaganfall	0,93 (0,82–1,05)	1,01 (0,78–1,30)
Alkoholkonsum oberhalb des tolerablen Grenzwerts	1,01 (0,89–1,14)	0,94 (0,71–1,23)
Depression	1,18 (0,96–1,45)	1,00 (0,66–1,49)
Angststörung	1,20 (0,97–1,46)	*1,64 (1,11–2,38)*
Schlafstörung	*1,25 (1,10–1,43)*	*1,53 (1,17–2,00)*
Körperliche Aktivität	1,00 (0,99–1,02)	1,00 (0,97–1,03)
Koronare Herzkrankheit	1,07 (0,77–1,48)	0,49 (0,19–1,08)
Periphere arterielle Verschlusskrankheit	*1,44 (1,08–1,88)*	1,35 (0,72–2,30)
Herzinfarkt	0,86 (0,56–1,29)	1,26 (0,48–2,84)
Herzinsuffizienz	0,95 (0,78–1,15)	1,25 (0,83–1,82)
Schlaganfall	0,81 (0,50–1,25)	0,38 (0,06–1,20)
Vorhofflimmern	*1,23 (1,08–1,40)*	*1,49 (1,15–1,93)*
*Beim Schlafen*		
Weibliches Geschlecht	*0,67 (0,53–0,84)*	1,04 (0,60–1,82)
Alter	*0,83 (0,78–0,89)*	*0,78 (0,66–0,91)*
Sozioökonomischer Status	0,99 (0,97–1,02)	1,00 (0,94–1,06)
Dauer des aktuellen Wohnsitzes	1,03 (0,98–1,08)	1,09 (0,99–1,21)
Nachtschichtarbeit	1,26 (0,99–1,58)	1,25 (0,68–2,20)
Diabetes mellitus	1,02 (0,65–1,52)	1,14 (0,42–2,63)
Bluthochdruck	0,90 (0,71–1,14)	1,13 (0,64–2,00)
Rauchen	0,76 (0,58–1,00)	0,85 (0,42–1,58)
Adipositas	1,16 (0,89–1,49)	1,14 (0,62–2,02)
Dyslipidämie	0,92 (0,73–1,16)	1,40 (0,81–2,44)
Familiengeschichte von Herzinfarkt oder Schlaganfall	0,97 (0,75–1,25)	0,76 (0,38–1,38)
Alkoholkonsum oberhalb des tolerablen Grenzwerts	1,04 (0,80–1,34)	0,62 (0,28–1,21)
Depression	1,23 (0,79–1,86)	0,61 (0,22–1,53)
Angststörung	0,86 (0,54–1,34)	1,65 (0,64–3,76)
Körperliche Aktivität	1,01 (0,98–1,04)	0,98 (0,91–1,05)
Schlafstörung	*1,40 (1,05–1,83)*	*2,44 (1,35–4,26)*
Koronare Herzkrankheit	1,24 (0,61–2,33)	0,79 (0,15–3,01)
Periphere arterielle Verschlusskrankheit	1,15 (0,59–2,04)	0,49 (0,03–2,32)
Herzinfarkt	0,79 (0,30–1,82)	2,08 (0,39–8,01)
Herzinsuffizienz	1,00 (0,65–1,48)	1,73 (0,73–3,55)
Schlaganfall	1,00 (0,35–2,24)	0,00 (0,00–32,00)
Vorhofflimmern	1,18 (0,88–1,54)	1,78 (0,98–3,11)
**Nachbarschaftslärm**		
*Am Tag*		
Weibliches Geschlecht	0,94 (0,87–1,02)	*1,28 (1,07–1,52)*
Alter	*0,83 (0,81–0,85)*	*0,90 (0,86–0,95)*
Sozioökonomischer Status	1,01 (1,00–1,01)	*0,93 (0,91–0,95)*
Dauer des aktuellen Wohnsitzes	*0,98 (0,96–0,99)*	*0,96 (0,93–1,00)*
Nachtschichtarbeit	*1,09 (1,00–1,19)*	*1,23 (1,02–1,49)*
Diabetes mellitus	1,02 (0,89–1,17)	1,02 (0,75–1,36)
Bluthochdruck	0,99 (0,92–1,08)	0,90 (0,76–1,08)
Rauchen	0,93 (0,85–1,02)	0,99 (0,81–1,20)
Adipositas	1,09 (0,99–1,19)	1,20 (1,00–1,45)
Dyslipidämie	0,98 (0,90–1,06)	1,03 (0,86–1,22)
Familiengeschichte von Herzinfarkt oder Schlaganfall	0,96 (0,88–1,04)	0,83 (0,68–1,00)
Alkoholkonsum oberhalb des tolerablen Grenzwerts	1,03 (0,95–1,13)	1,08 (0,89–1,31)
Depression	*1,38 (1,18–1,62)*	*1,59 (1,19–2,10)*
Angststörung	*1,26 (1,07–1,47)*	*1,33 (1,00–1,77)*
Schlafstörung	*1,14 (1,03–1,25)*	*1,24 (1,01–1,51)*
Körperliche Aktivität	0,99 (0,98–1,00)	1,00 (0,98–1,02)
Koronare Herzkrankheit	1,07 (0,86–1,34)	1,14 (0,68–1,82)
Periphere arterielle Verschlusskrankheit	1,05 (0,85–1,30)	0,89 (0,54–1,40)
Herzinfarkt	0,98 (0,74–1,28)	0,64 (0,31–1,23)
Herzinsuffizienz	0,93 (0,81–1,07)	0,90 (0,64–1,23)
Schlaganfall	1,02 (0,76–1,36)	1,05 (0,53–1,87)
Vorhofflimmern	*1,26 (1,14–1,38)*	*1,58 (1,31–1,89)*
*Beim Schlafen*		
Weibliches Geschlecht	1,08 (0,97–1,19)	*1,53 (1,23–1,92)*
Alter	*0,83 (0,80–0,85)*	*0,91 (0,85–0,96)*
Sozioökonomischer Status	*0,99 (0,98–1,00)*	*0,96 (0,93–0,98)*
Dauer des aktuellen Wohnsitzes	0,99 (0,97–1,01)	*0,94 (0,89–0,98)*
Nachtschichtarbeit	1,10 (0,99–1,23)	*1,31 (1,03–1,65)*
Diabetes mellitus	1,11 (0,92–1,32)	1,08 (0,73–1,55)
Bluthochdruck	1,00 (0,90–1,11)	0,99 (0,79–1,24)
Rauchen	*0,82 (0,73–0,92)*	0,96 (0,75–1,22)
Adipositas	1,04 (0,93–1,17)	1,20 (0,94–1,51)
Dyslipidämie	1,10 (0,99–1,22)	1,12 (0,89–1,39)
Familiengeschichte von Herzinfarkt oder Schlaganfall	0,98 (0,87–1,09)	1,01 (0,80–1,28)
Alkoholkonsum oberhalb des tolerablen Grenzwerts	1,14 (1,02–1,27)	1,19 (0,93–1,51)
Depression	*1,30 (1,08–1,57)*	1,37 (0,96–1,94)
Angststörung	*1,23 (1,02–1,48)*	1,36 (0,95–1,91)
Schlafstörung	*1,44 (1,28–1,62)*	*1,54 (1,20–1,96)*
Körperliche Aktivität	0,99 (0,98–1,01)	1,00 (0,97–1,03)
Koronare Herzkrankheit	0,89 (0,64–1,21)	0,91 (0,46–1,67)
Periphere arterielle Verschlusskrankheit	1,22 (0,93–1,59)	1,27 (0,73–2,09)
Herzinfarkt	1,03 (0,70–1,49)	1,12 (0,50–2,29)
Herzinsuffizienz	1,05 (0,88–1,26)	1,02 (0,67–1,47)
Schlaganfall	1,14 (0,77–1,65)	0,61 (0,18–1,46)
Vorhofflimmern	*1,26 (1,12–1,42)*	*1,61 (1,28–2,02)*

Odds Ratios (*OR*) und 95 %-Konfidenzintervalle (*95* *%-KI*) wurden mittels multivariabler logistischer Regressionsanalysen bestimmt mit der Lärmbelästigung als abhängige (wenig, mittel, stark und äußerst vs. überhaupt nicht sowie überhaupt nicht, wenig, mittel vs. stark und äußerst) und den Determinanten als unabhängige Variablen. Alter und Dauer des aktuellen Wohnsitzes wurden angegeben pro Zunahme um 5 Jahre. Körperliche Aktivität wurde angegeben pro Zunahme um 1 Einheit. Relevante Assoziationen wurden *kursiv* hervorgehoben

## Diskussion

### Relevante Quellen der Lärmbelästigung in der Bevölkerung

Die subjektive Lärmbelästigung wird als komplexes, mehrdimensionales Konstrukt betrachtet, das in seiner Gesamtheit die negative psychische Repräsentation einer Person in Bezug auf eine Lärmquelle bzw. Lärmexposition widerspiegelt [18]. Nach Angaben der WHO ist Lärmbelästigung ein Indikator der Gesundheitsbelastung in der Bevölkerung, die nach Schlafstörungen die zweitwichtigste gesundheitliche Konsequenz von Umgebungslärm darstellt [2]. In der vorliegenden Population war Lärmbelästigung ein weit verbreitetes Phänomen mit einem fast epidemischen Charakter, von dem etwa 80 % der Probanden betroffen waren. Etwa ein Drittel der Probanden berichtete sogar über starke und äußerste Lärmbelästigung. Dominierende Quellen der Lärmbelästigung stellten Flug‑, Straßenverkehrs- und Nachbarschaftslärmbelästigung dar. Unsere Ergebnisse stehen im Einklang mit einer repräsentativen Erhebung des Umweltbundesamts, in der sich etwa 89 % der deutschen Bevölkerung durch unterschiedliche Lärmquellen belästigt fühlten, wobei hier Straßenverkehrs- (75 %), Nachbarschafts- (60 %) und Industrielärm (42 %) dominierten [19]. In der NAKO(ehemals: Nationale Kohorte)-Gesundheitsstudie fühlten sich dagegen nahezu zwei Drittel der Probanden nicht durch Lärm belästigt, wobei hier lediglich die nächtliche Verkehrslärmbelästigung untersucht wurde, die in der vorliegenden Studie ebenfalls geringer ausfiel [20]. In 2 Erhebungen des Robert Koch-Instituts berichtet etwa die Hälfte der Probanden, durch Lärm in ihrem Wohnumfeld belästigt zu werden (Gesundheit in Deutschland aktuell [GEDA]; [21]), wobei sich laut den Daten des DEGS1 (Studie zur Gesundheit Erwachsener in Deutschland, Erhebungswelle 1) 37,4 % der Probanden durch Straßenverkehrslärm belästigt fühlten [22]. Da das Studiengebiet der GHS Teile des stark fluglärmexponierten Rhein-Main-Gebiets durch den Flughafen Frankfurt am Main umschließt, ist der Fluglärm in der vorliegenden Untersuchung stärker vertreten. Interessanterweise konnten Babisch et al. anhand Daten der HYENA(Hypertension and Exposure to Noise near Airports)-Studie demonstrieren, dass die Fluglärmbelästigung im Gegensatz zur Straßenlärmbelästigung in den letzten Jahren drastisch zugenommen hat und dass bei identischen Lärmpegeln der Fluglärm zur stärksten Belästigungsreaktion führt, gefolgt von Straßen- und Schienenverkehrslärm [23, 24]. Dies könnte sowohl an der spezifischen Geräuschcharakteristik (intermittierender, zyklischer Charakter mit schnell ansteigenden und absteigenden Lärmpegeln, Lärmfrequenz) als auch an einer negativen Einstellung gegenüber dem Flugverkehr liegen [25].

### Lärmbelästigung, Begleiterkrankungen und weitere Determinanten

Angesichts des hohen Grads an Lärmbelästigung in der Bevölkerung und der Bedeutsamkeit hinsichtlich der Gesundheit ist die Bestimmung von Determinanten der Lärmbelästigung relevant. Wichtig ist anzumerken, dass objektiv messbare Lärmpegel nur etwa bis zu ein Drittel der Unterschiede in der subjektiven Lärmbelästigung erklären [26]. Neben objektiven bzw. akustischen Faktoren der Lärmexposition wie Intensität, Frequenz, Komplexität, Dauer, Zeitpunkt und Anzahl der Lärmereignisse wird die Lärmbelästigung v. a. durch individuelle, soziale und situative Faktoren bestimmt [27–29]. Entsprechend früheren Untersuchungen, war in der vorliegenden Analyse das Vorhandensein einer Depression, Angststörung und Schlafstörung mit erhöhter Lärmbelästigung assoziiert [16, 30]. Der substanzielle Zusammenhang zwischen Lärm/Lärmbelästigung und psychischer Gesundheit war Gegenstand aktueller Übersichtsarbeiten [5, 31]. Insbesondere der Zusammenhang mit Schlafstörungen, die einen wesentlichen pathophysiologischen Mechanismus bei der Vermittlung lärminduzierter Erkrankungen darstellen, war stark ausgeprägt [6]. Hier scheint ein zu kurzer und häufig unterbrochener Schlaf einen wichtigen Stimulus für Störungen der zirkadianen Rhythmik und, damit verbunden, des oxidativen Stresses in Gefäßen und Gehirn sowie der Gefäßfunktion (Endothelfunktion) darzustellen [6]. In Bezug auf kardiovaskuläre Erkrankungen war Vorhofflimmern im Einklang mit einer früheren Untersuchung mit erhöhter Lärmbelästigung assoziiert [17]. Dagegen war der Risikofaktor Rauchen, der möglicherweise eine maladaptive Bewältigungsstrategie im Umgang mit Lärmstress darstellt, tendenziell mit niedrigerer Lärmbelästigung verbunden. Hinsichtlich des Alters legen im Einklang mit den vorliegenden Ergebnissen weitere Untersuchungen nahe, dass ein kurvilinearer Zusammenhang mit der Lärmbelästigung besteht [20, 32], die mit zunehmendem Alter abnimmt und im mittleren Altersbereich am stärksten ausgeprägt ist [28, 33, 34]. Dass die Prävalenz von Lärmbelästigung bei Frauen eher geringer ausfiel, stellt möglicherweise eine Erklärung für die in verschiedenen Studien gefundenen geschlechtsspezifischen Unterschiede für den Zusammenhang zwischen der Verkehrslärmexposition und kardiovaskulären Endpunkten wie Bluthochdruck und der Einnahme von Antihypertonika dar, die auf einen stärkeren Zusammenhang bei Männern hinweisen [35–38]. Der Zusammenhang mit dem sozioökonomischen Status ist als weniger konsistent zu bewerten. Hier ist denkbar, dass Lärmbelästigung sowohl sozioökonomisch schwächere [39–41] als auch stärkere Bevölkerungsschichten betrifft [42, 43]. Die Vergleichbarkeit zwischen Studien wird jedoch durch den Einsatz verschiedener Messinstrumente zur Bestimmung des sozioökonomischen Status erschwert.

### Limitationen

Wesentliche Stärken der vorliegenden Studie umfassen die große, bevölkerungsrepräsentative Stichprobe sowie die standardisierte Erfassung der Lärmbelästigung durch verschiedene Quellen am Tag und beim Schlafen. Der Einsatz multivariabler Regressionsmodelle mit wechselseitiger Kontrolle aller eingeschlossenen Variablen wirkt verzerrenden Einflüssen entgegen und sichert somit hohe interne Validität. Eine bedeutsame Einschränkung der Ergebnisse ist das Fehlen von objektiv messbaren Lärmpegeln, die einen wesentlichen Einfluss auf die Lärmbelästigung haben. Die Ermittlung von objektiv messbaren Lärmpegeln in der GHS ist jedoch in Zukunft geplant. Auch ist die Direktionalität der Ergebnisse aufgrund des Querschnittdesigns der Untersuchung nur eingeschränkt bewertbar. Zuletzt sollte erwähnt werden, dass die Generalisierbarkeit der Ergebnisse durch die Gegebenheit einer stärkeren Repräsentation von Fluglärm(-belästigung) in der vorliegenden Population eingeschränkt wird.

## Fazit für die Praxis

- Die European Environment Agency (EEA) geht davon aus, dass Lärmbelästigung einen Großteil der Bevölkerung betrifft, wobei insbesondere der Flug‑, Straßen- und Schienenverkehr für Lärmbelästigung bei 53 Mio. Erwachsenen in Europa sorgt.
- Ergebnisse früherer Studien zeigen, dass dauerhaft erhöhte Lärmpegel persistierende Lärmbelästigungs- bzw. Stressreaktionen auslösen können, die mit erhöhter kardiovaskulärer Morbidität und Mortalität assoziiert sind.
- In der vorliegenden Studie fühlten sich etwa 80 % der Probanden durch Lärm belästigt. Dominierende Quellen der Belästigung waren Flug‑, Straßenverkehrs- und Nachbarschaftslärm.
- Relevante Determinanten der Lärmbelästigung umfassten soziodemographische Variablen, kardiovaskuläre Risikofaktoren sowie Erkrankungen.
- Präventive Maßnahmen sind zwingend erforderlich, um die Bevölkerung vor Lärmbelästigung und assoziierten gesundheitlichen Konsequenzen zu schützen.

## Supplementary Information




