# Supplementary material for: Noise annoyance in the German general population: Prevalence and determinants in the Gutenberg Health Study
Source: Herz. 2021 Aug 13;47(3):265–79. [Article in German] doi: 10.1007/s00059-021-05060-z (PMC9205798; doi:10.1007/s00059-021-05060-z)
Supplement: Supplementary file 1 [file 59_2021_5060_MOESM1_ESM.docx]

**E-Supplement**

**Lärmbelästigung in der deutschen Allgemeinbevölkerung – Prävalenz und Determinanten in der Gutenberg-Gesundheitsstudie**

Dr. rer. physiol. Omar Hahad^1,2^*, Univ.-Prof. Dr. med. Manfred Beutel^3^, Univ.-Prof. Dr. med. Matthias Michal^2,3^, Dr. rer. physiol. Andreas Schulz^4^, Univ.-Prof. Dr. med. Norbert Pfeiffer^5^, Dr. rer. physiol. Emilio Gianicolo^6^, Univ.-Prof. Dr. med. Karl Lackner^2,7^, Univ.-Prof. Dr. med. Philipp Wild^2,4^, Univ.-Prof. Dr. rer. nat. Andreas Daiber^1,2^, Univ.-Prof. Dr. med. Thomas Münzel^1,2^

^1^ Zentrum für Kardiologie – Kardiologie I, Universitätsmedizin der Johannes Gutenberg-Universität Mainz, Deutschland

^2^ Deutsches Zentrum für Herz-Kreislauf-Forschung (DZHK), Standort Rhein-Main, Mainz, Deutschland

^3^ Klinik und Poliklinik für Psychosomatische Medizin und Psychotherapie, Universitätsmedizin der Johannes Gutenberg-Universität Mainz, Deutschland

^4^ Zentrum für Kardiologie – Präventive Kardiologie und Medizinische Prävention, Universitätsmedizin der Johannes Gutenberg-Universität Mainz, Deutschland

^5^ Augenklinik und Poliklinik, Universitätsmedizin der Johannes Gutenberg-Universität Mainz, Deutschland

^6^ Institut für Medizinische Biometrie, Epidemiologie und Informatik (IMBEI), Universitätsmedizin der Johannes Gutenberg-Universität Mainz, Deutschland

^7^ Institut für Klinische Chemie und Laboratoriumsmedizin, Universitätsmedizin der Johannes Gutenberg-Universität Mainz, Deutschland

***Korrespondenzadresse:**

Dr. rer. physiol. Omar Hahad

Universitätsmedizin der Johannes Gutenberg-Universität Mainz

Zentrum für Kardiologie – Kardiologie I

Langenbeckstraße 1, 55131 Mainz, Deutschland

Tel.: +49 (0) 6131 17-7250

Fax: +49 (0) 6131 17-6615

E-Mail: [tmuenzel@uni-mainz.de](mailto:tmuenzel@uni-mainz.de) / omar.hahad@unimedizin-mainz.de

**Tabelle e1. Angaben zur Lärmbelästigung.**

| **Lärmquelle** | | **Tagsüber** | | | **Beim Schlafen** | |
| --- | --- | --- | --- | --- | --- | --- |
| Straßenverkehrslärm | | „Wie stark fühlen Sie sich tagsüber in den letzten Jahren durch Straßenverkehrslärm belästigt?“ | | | „Wie stark fühlen Sie sich beim Schlafen in den letzten Jahren durch Straßenverkehrslärm belästigt?“ | |
| Schienenverkehrslärm | | „Wie stark fühlen Sie sich tagsüber in den letzten Jahren durch Schienenverkehrslärm belästigt?“ | | | „Wie stark fühlen Sie sich beim Schlafen in den letzten Jahren durch Schienenverkehrslärm belästigt?“ | |
| Fluglärm | | „Wie stark fühlen Sie sich tagsüber in den letzten Jahren durch Fluglärm belästigt?“ | | | „Wie stark fühlen Sie sich beim Schlafen in den letzten Jahren durch Fluglärm belästigt?“ | |
| Industrie-, Bau- und Gewerbelärm | | „Wie stark fühlen Sie sich tagsüber in den letzten Jahren durch Industrie-, Bau- oder Gewerbelärm belästigt?“ | | | „Wie stark fühlen Sie sich beim Schlafen in den letzten Jahren durch Industrie-, Bau- oder Gewerbelärm belästigt?“ | |
| Nachbarschaftslärm | | „Wie stark fühlen Sie sich tagsüber in den letzten Jahren durch Lärm im Haus belästigt?“ | | | „Wie stark fühlen Sie sich beim Schlafen in den letzten Jahren durch Lärm im Haus belästigt?“ | |
| **Antwortformat** | | | | | | |
| „0 = Überhaupt nicht“ | „1 = Wenig“ | | „2 = Mittel“ | „3 = Stark“ | | „4 = Äußerst“ |

Die Angaben zur Lärmbelästigung wurden im Rahmen des computergestützten persönlichen Interviews erhoben.

**Tabelle e2. Definition von soziodemographischen Variablen, kardiovaskulären Risikofaktoren und Erkrankungen.**

| **Variable** | **Definition** |
| --- | --- |
| Sozioökonomischer Status | Für die Ermittlung des sozioökonomischen Status wurde der validierte SES-Index von Lampert und Kroll verwendet (1). Der SES-Index bewertet Informationen zum Bildungsstand, Berufsstand und Einkommen. Resultierend ergibt sich eine Summenwert zwischen 3 und 21, wobei ein höherer Summenwert einem höheren sozioökonomischen Status entspricht. |
| Nachtschichtarbeit | Wurde definiert als Arbeitszeiten zwischen 23 und 5 Uhr in den letzten Monaten. |
| Diabetes mellitus | Lag bei der ärztlichen Diagnose eines Diabetes mellitus oder bei der Einnahme von blutzuckersenkenden Medikamenten oder einer Insulintherapie vor. Zudem wurden Probanden als Diabetiker klassifiziert, wenn ein nüchtern (Nüchternzeit von mindestens 8 Stunden) Blutzuckerspiegel von ≥ 126 mg/dl oder ein HbA1c von > 6,5% oder ein Blutzuckerspiegel bei weniger als 8 Stunden Nüchternzeit von ≥ 200 mg/dl vorlag. |
| Bluthochdruck | Der Blutdruck wurde mittels einer Blutdruckmanschette am linken Oberarm in sitzender Position und nach einer Ruhephase von mindestens fünf Minuten gemessen. Bluthochdruck wurde definiert als systolischer Blutdruck ≥ 140 mmHg oder als diastolischer Blutdruck ≥ 90 mmHg (bestimmt durch den Mittelwert der 2. und 3. standardisierten Messung nach einer Ruhezeit von 8 und 11 Minuten). Eine Einnahme von blutdrucksenkenden Medikamenten innerhalb der letzten zwei Wochen oder die ärztliche Diagnose eines Bluthochdrucks wurde ebenfalls für das Vorliegen eines Bluthochdrucks herangezogen. |
| Rauchen | Wurde kategorisiert als nie geraucht, ehemalig geraucht und aktuell Rauchen. Als aktuelle Raucher galten die Probanden, die täglich bzw. regelmäßig geraucht haben, d. h. mindestens eine Zigarette pro Tag, sieben Zigaretten pro Woche oder eine Packung pro Monat in den vergangen sechs Monaten. Probanden, die nie täglich bzw. regelmäßig geraucht haben, galten als Nichtraucher. Als ehemalige Raucher galten die Probanden, die für mindestens sechs Monate nicht mehr geraucht haben. Ehemalige Raucher und Probanden, die nie geraucht haben, wurden als Nichtraucher klassifiziert, wobei aktuelle Raucher als Raucher klassifiziert wurden. |
| Adipositas | Lag ab einem Body-Mass-Index von 30 vor. Die Berechnung des Body-Mass-Index ergab sich aus der Division des Körpergewichtes in Kilogramm durch die Körpergröße in Meter zum Quadrat. Die Körpergröße wurde als maximale Distanz vom höchsten Punkt am Kopf bis zu den Füßen gemessen während die Probanden barfuß waren. Das Körpergewicht wurde mit einer Digitalwaage gemessen. |
| Dyslipidämie | Wurde definiert als ärztliche Diagnose einer Dyslipidämie, LDL / HDL-Verhältnis von > 3,5 oder Triglycerid-Spiegel von ≥ 150 mg/dl. |
| Familiengeschichte von Herzinfarkt oder Schlaganfall | Lag bei dem Auftreten bei einem weiblichen Verwandten ersten Grades von ≤ 65 Jahren oder bei einem männlichen Verwandten ersten Grades von ≤ 60 Jahren vor. |
| Alkoholkonsum | Wurde definiert als Alkoholkonsum unterhalb und oberhalb des tolerablen Grenzwerts (> 24 g pro Tag für Männer und > 12 g pro Tag für Frauen) (2). |
| Depression | Wurde anhand des Depressionsmoduls des Gesundheitsbogens für Patienten (PHQ-9, Summenwert zwischen 0 und 27) definiert, wobei ein Summenwert ≥ 10 auf das Vorhandensein einer Depression hinweist (3). |
| Angststörung | Wurde anhand des Angstmoduls des Gesundheitsbogens für Patienten (GAD-2, Summenwert zwischen 0 und 6) definiert, wobei ein Summenwert ≥ 3 auf das Vorhandensein einer generalisierten Angststörung hinweist (4). |
| Schlafstörung | Wurde anhand eines Items des Depressionsmoduls des Gesundheitsbogens für Patienten (PHQ-9, „Schwierigkeiten ein- oder durchzuschlafen oder vermehrter Schlaf“) definiert. Die Probanden wurden gebeten auf Basis einer vierstufigen Likert-Skala anzugeben: „Wie oft fühlten Sie sich im Verlauf der letzten 2 Wochen durch die folgenden Beschwerden beeinträchtigt? Eine klinisch relevante Schlafstörung lag vor, sofern die Probanden mindestens angaben: „An mehr als der Hälfte der Tage“ (5). |
| Körperliche Aktivität | Wurde mittels eines validierten Fragebogens (Short Questionnaire to Assess Health-Enhancing Physical Activity – SQUASH) bestimmt, der Angaben zu verschiedenen Aktivitäten enthält im Rahmen einer normalen Woche in den letzten Monaten, um einen Indexwert zu berechnen (Gesamtminuten der Aktivität multipliziert mit Intensitätswert multipliziert mit dem Faktor 1000) (6). |
| Kardiovaskuläre Erkrankungen | Wurde anamnestisch oder anhand der Diagnose während den Studienuntersuchungen bestimmt. Kardiovaskuläre Erkrankungen umfassten koronare Herzerkrankung, periphere arterielle Verschlusskrankheit, Herzinfarkt, Herzinsuffizienz, Schlaganfall und Vorhofflimmern. |

**Tabelle e3. Prävalenz von Lärmbelästigung stratifiziert nach Geschlecht.**

| **Lärmbelästigung** | **Männer** | **Frauen** | **Gesamt** |
| --- | --- | --- | --- |
| **Am Tag (> 0)** | | | |
| Straßenverkehrslärm | 42,3 (3 132) | 40,1 (2 903) | 41,2 (6 035) |
| Fluglärm | 60,7 (4 492) | 56,0 (4 052) | 58,4 (8 544) |
| Schienenverkehrslärm | 15,5 (1 148) | 13,5 (975) | 14,5 (2 123) |
| Industrie-, Bau- und Gewerbelärm | 14,3 (1 055) | 12,6 (912) | 13,5 (1 967) |
| Nachbarschaftslärm | 36,3 (2 684) | 35,7 (2 579) | 36,0 (5 263) |
| **Beim Schlafen (> 0)** | | | |
| Straßenverkehrslärm | 16,2 (1 198) | 16,5 (1 192) | 16,4 (2 390) |
| Fluglärm | 32,9 (2 429) | 30,1 (2 170) | 31,5 (4 599) |
| Schienenverkehrslärm | 8,7 (642) | 7,4 (536) | 8,1 (1 178) |
| Industrie-, Bau- und Gewerbelärm | 3,0 (225) | 2,2 (156) | 2,6 (381) |
| Nachbarschaftslärm | 15,4 (1 139) | 17,1 (1 234) | 16,3 (2 373) |
| **Totale Lärmbelästigung (> 0)** | 80,2 (5 939) | 78,5 (5 676) | 79,3 (11 615) |

Dargestellt als relative und absolute Häufigkeiten.

**Tabelle e4. Prävalenz von Lärmbelästigung stratifiziert nach Geschlecht und kardiovaskulären Erkrankungen.**

| **Lärmbelästigung** | **Männer** | | **Frauen** | | **Gesamt** | |
| --- | --- | --- | --- | --- | --- | --- |
|  | *Koronare Herzerkrankung* | | | | | |
|  | *Nein* | *Ja* | *Nein* | *Ja* | *Nein* | *Ja* |
| **Am Tag (> 0)** | | | | | | |
| Straßenverkehrslärm | 42,2 (2 885) | 44,2 (201) | 40,2 (2 817) | 40,8 (58) | 41,2 (5 702) | 43,4 (259) |
| Fluglärm | 60,7 (4 147) | 60,9 (277) | 56,3 (3 947) | 50,7 (72) | 58,5 (8 094) | 58,5 (349) |
| Schienenverkehrslärm | 15,5 (1 057) | 15,6 (71) | 13,4 (941) | 16,2 (23) | 14,5 (1 998) | 15,8 (94) |
| Industrie-, Bau- und Gewerbelärm | 14,4 (984) | 11,0 (50) | 12,7 (889) | 9,9 (14) | 13,5 (1 873) | 10,7 (64) |
| Nachbarschaftslärm | 36,7 (2 505) | 29,9 (136) | 35,9 (2 519) | 26,8 (38) | 36,3 (5 024) | 29,1 (174) |
| **Beim Schlafen (> 0)** | | | | | | |
| Straßenverkehrslärm | 16,3 (1 108) | 16,0 (72) | 16,5 (1 157) | 14,2 (20) | 16,4 (2 265) | 15,5 (92) |
| Fluglärm | 32,8 (2 233) | 34,8 (157) | 30,3 (2 121) | 23,0 (32) | 31,5 (4 354) | 32,0 (189) |
| Schienenverkehrslärm | 8,8 (598) | 8,4 (38) | 7,3 (513) | 11,5 (16) | 8,1 (1 111) | 9,2 (54) |
| Industrie-, Bau- und Gewerbelärm | 3,1 (212) | 2,2 (10) | 2,2 (152) | 2,9 (4) | 2,6 (364) | 2,4 (14) |
| Nachbarschaftslärm | 15,6 (1 065) | 12,4 (56) | 17,2 (1 205) | 11,4 (16) | 16,4 (2 270) | 12,2 (72) |
| **Totale Lärmbelästigung (> 0)** | 80,2 (5 478) | 80,9 (368) | 78,7 (5 517) | 76,1 (108) | 79,4 (10 995) | 79,7 (476) |
|  | *Herzinfarkt* | | | | | |
|  | *Nein* | *Ja* | *Nein* | *Ja* | *Nein* | *Ja* |
| **Am Tag (> 0)** | | | | | | |
| Straßenverkehrslärm | 42,5 (2 991) | 38,0 (124) | 40,1 (2 859) | 42,6 (40) | 41,3 (5 850) | 39,0 (164) |
| Fluglärm | 61,1 (4 303) | 53,1 (173) | 56,2 (4 006) | 41,5 (39) | 58,7 (8 309) | 50,5 (212) |
| Schienenverkehrslärm | 15,5 (1 092) | 15,6 (51) | 13,4 (954) | 20,4 (19) | 14,5 (2 046) | 16,7 (70) |
| Industrie-, Bau- und Gewerbelärm | 14,5 (1 019) | 9,5 (31) | 12,6 (897) | 11,7 (11) | 13,5 (1 916) | 10,0 (42) |
| Nachbarschaftslärm | 36,5 (2 571) | 30,1 (98) | 35,8 (2 550) | 25,5 (24) | 36,2 (5 121) | 29,0 (122) |
| **Beim Schlafen (> 0)** | | | | | | |
| Straßenverkehrslärm | 16,4 (1 154) | 11,7 (38) | 16,5 (1 173) | 16,1 (15) | 16,5 (2 327) | 12,7 (53) |
| Fluglärm | 33,1 (2 324) | 29,2 (95) | 30,2 (2 149) | 18,7 (17) | 31,7 (4 473) | 26,9 (112) |
| Schienenverkehrslärm | 8,7 (610) | 8,6 (28) | 7,3 (521) | 14,3 (13) | 8,0 (1 131) | 9,9 (41) |
| Industrie-, Bau- und Gewerbelärm | 3,1 (221) | 1,2 (4) | 2,1 (151) | 4,4 (4) | 2,6 (372) | 1,9 (8) |
| Nachbarschaftslärm | 15,6 (1 094) | 11,7 (38) | 17,1 (1 218) | 14,1 (13) | 16,4 (2 312) | 12,2 (51) |
| **Totale Lärmbelästigung (> 0)** | 80,4 (5 666) | 75,8 (247) | 78,6 (5 601) | 70,2 (66) | 79,5 (1 11 267) | 74,5 (313) |
|  | *Schlaganfall* | | | | | |
|  | *Nein* | *Ja* | *Nein* | *Ja* | *Nein* | *Ja* |
| **Am Tag (> 0)** | | | | | | |
| Straßenverkehrslärm | 42,5 (3 055) | 35,3 (59) | 40,2 (2 867) | 36,1 (30) | 41,3 (5 922) | 35,6 (89) |
| Fluglärm | 61,05 (4 385) | 50,9 (85) | 56,3 (4 012) | 41,0 (34) | 58,6 (8 397) | 47,6 (119) |
| Schienenverkehrslärm | 15,5 (1 111) | 16,2 (27) | 13,5 (964) | 10,8 (9) | 14,5 (2 075) | 14,4 (36) |
| Industrie-, Bau- und Gewerbelärm | 14,3 (1 028) | 10,8 (18) | 12,7 (905) | 7,2 (6) | 13,5 (1 933) | 9,6 (24) |
| Nachbarschaftslärm | 36,5 (2 621) | 28,1 (47) | 35,7 (2 547) | 33,7 (28) | 36,1 (5 168) | 30,0 (75) |
| **Beim Schlafen (> 0)** | | | | | | |
| Straßenverkehrslärm | 16,3 (1 169) | 13,4 (22) | 16,5 (1 176) | 15,7 (13) | 16,4 (2 345) | 14,2 (35) |
| Fluglärm | 33,0 (2 372) | 28,7 (47) | 30,3 (2 152) | 18,1 (15) | 31,7 (4 524) | 25,1 (62) |
| Schienenverkehrslärm | 8,6 (620) | 9,8 (16) | 7,4 (527) | 8,4 (7) | 8,0 (1 147) | 9,3 (23) |
| Industrie-, Bau- und Gewerbelärm | 3,1 (219) | 2,4 (4) | 2,2 (153) | 3,6 (3) | 2,6 (372) | 2,8 (7) |
| Nachbarschaftslärm | 15,5 (1 110) | 13,4 (22) | 17,1 (1 218) | 18,1 (15) | 16,3 (2 328) | 15,0 (37) |
| **Totale Lärmbelästigung (> 0)** | 80,3 (5 777) | 76,0 (127) | 78,6 (5 606) | 69,9 (58) | 79,4 (11 383) | 74,0 (185) |
|  | *Vorhofflimmern* | | | | | |
|  | *Nein* | *Ja* | *Nein* | *Ja* | *Nein* | *Ja* |
| **Am Tag (> 0)** | | | | | | |
| Straßenverkehrslärm | 42,0 (2 695) | 44,4 (436) | 39,4 (2 217) | 42,7 (686) | 40,8 (4 912) | 43,3 (1 122) |
| Fluglärm | 60,5 (3 881) | 62,2 (610) | 55,5 (3 119) | 58,1 (933) | 58,1 (7 000) | 59,6 (1 543) |
| Schienenverkehrslärm | 15,2 (976) | 17,5 (172) | 13,5 (757) | 13,6 (218) | 14,4 (1 733) | 15,1 (390) |
| Industrie-, Bau- und Gewerbelärm (%) | 14,1 (905) | 15,3 (150) | 12,1 (682) | 14,3 (230) | 13,2 (1 587) | 14,7 (380) |
| Nachbarschaftslärm (%) | 36,0 (2312) | 37,9 (372) | 34,5 (1 939) | 39,9 (640) | 35,3 (4 251) | 39,1 (1 012) |
| **Beim Schlafen (> 0)** | | | | | | |
| Straßenverkehrslärm | 16,0 (1 027) | 17,5 (171) | 15,6 (873) | 19,9 (319) | 15,8 (1 900) | 19,0 (490) |
| Fluglärm | 32,3 (2 068) | 36,9 (361) | 29,2 (1 641) | 33,1 (529) | 30,9 (3 709) | 34,5 (890) |
| Schienenverkehrslärm | 8,4 (540) | 10,4 (102) | 7,1 (400) | 8,5 (136) | 7,8 (940) | 9,2 (238) |
| Industrie-, Bau- und Gewerbelärm | 3,0 (195) | 3,1 (30) | 2,1 (117) | 2,4 (39) | 2,6 (312) | 2,7 (69) |
| Nachbarschaftslärm | 15,4 (988) | 15,4 (151) | 15,8 (887) | 21,7 (347) | 15,6 (1 875) | 19,3 (498) |
| **Totale Lärmbelästigung (> 0) (%)** | 79,7 (5 118) | 83,4 (819) | 77,3 (4 347) | 82,7 (1 329) | 78,6 (9 465) | 83,0 (2 148) |
|  | *Periphere arterielle Verschlusskrankheit* | | | | | |
|  | *Nein* | *Ja* | *Nein* | *Ja* | *Nein* | *Ja* |
| **Am Tag (> 0)** | | | | | | |
| Straßenverkehrslärm | 42,4 (3 000) | 38,5 (99) | 40,1 (2 784) | 41,7 (90) | 41,2 (5 784) | 40,0 (189) |
| Fluglärm | 60,8 (4 299) | 58,4 (150) | 56,1 (3 891) | 57,9 (125) | 58,4 (8 190) | 58,1 (275) |
| Schienenverkehrslärm | 15,4 (1 092) | 18,0 (46) | 13,4 (931) | 15,3 (33) | 14,4 (2 023) | 16,7 (79) |
| Industrie-, Bau- und Gewerbelärm | 14,1 (999) | 16,7 (43) | 12,6 (877) | 12,0 (26) | 13,4 (1 876) | 14,6 (69) |
| Nachbarschaftslärm | 36,4 (2 575) | 31,5 (81) | 35,8 (2 483) | 32,9 (71) | 36,1 (5 058) | 32,1 (152) |
| **Beim Schlafen (> 0)** | | | | | | |
| Straßenverkehrslärm | 16,3 (1 149) | 14,8 (38) | 16,5 (1 146) | 18,1 (39) | 16,4 (2 295) | 16,3 (77) |
| Fluglärm | 33,0 (2 330) | 30,9 (79) | 30,2 (2 092) | 26,4 (57) | 31,6 (4 422) | 28,8 (136) |
| Schienenverkehrslärm | 8,7 (612) | 9,4 (24) | 7,4 (510) | 8,8 (19) | 8,0 (1 122) | 9,1 (43) |
| Industrie-, Bau- und Gewerbelärm | 3,0 (214) | 3,9 (10) | 2,2 (152) | 0,9 (2) | 2,6 (366) | 2,5 (12) |
| Nachbarschaftslärm | 15,4 (1 087) | 15,6 (40) | 17,1 (1 182) | 17,1 (37) | 16,2 (2 269) | 16,3 (77) |
| **Totale Lärmbelästigung (> 0)** | 80,2 (5 683) | 77,8 (200) | 78,7 (5 461) | 76,9 (166) | 79,5 (11 144) | 77,4 (366) |
|  | *Herzinsuffizienz* | | | | | |
|  | *Nein* | *Ja* | *Nein* | *Ja* | *Nein* | *Ja* |
| **Am Tag (> 0)** | | | | | | |
| Straßenverkehrslärm | 42,0 (2 811) | 45,0 (318) | 40,2 (2 745) | 39,2 (156) | 41,1 (5 556) | 42,9 (474) |
| Fluglärm | 61,2 (4 093) | 56,2 (397) | 56,1 (3 830) | 55,3 (220) | 58,6 (7 923) | 55,9 (617) |
| Schienenverkehrslärm | 15,4 (1 032) | 16,5 (116) | 13,4 (916) | 14,6 (58) | 14,4 (1 948) | 15,8 (174) |
| Industrie-, Bau- und Gewerbelärm | 14,4 (962) | 13,1 (92) | 12,7 (869) | 10,8 (43) | 13,5 (1 831) | 12,3 (135) |
| Nachbarschaftslärm | 36,5 (2 442) | 34,1 (240) | 35,9 (2 453) | 31,4 (125) | 36,2 (4 895) | 33,1 (365) |
| **Beim Schlafen (> 0)** | | | | | | |
| Straßenverkehrslärm | 16,1 (1 073) | 17,7 (124) | 16,7 (1 139) | 13,4 (53) | 16,4 (2 212) | 16,2 (177) |
| Fluglärm | 33,1 (2 210) | 31,2 (218) | 30,1 (2 050) | 30,1 (119) | 31,6 (4 260) | 30,8 (337) |
| Schienenverkehrslärm | 8,6 (574) | 9,7 (68) | 7,5 (508) | 6,8 (27) | 8,0 (1 082) | 8,7 (95) |
| Industrie-, Bau- und Gewerbelärm | 3,0 (203) | 3,2 (22) | 2,2 (150) | 1,5 (6) | 2,6 (353) | 2,6 (28) |
| Nachbarschaftslärm | 15,4 (1 030) | 15,5 (108) | 17,1 (1 167) | 16,9 (67) | 16,3 (2 197) | 16,0 (175) |
| **Totale Lärmbelästigung (> 0)** | 80,2 (5 375) | 79,5 (561) | 78,6 (5 368) | 76,6 (305) | 79,4 (10 743) | 78,4 (866) |

Dargestellt als relative und absolute Häufigkeiten.

**eLiteratur**

e1. Lampert T, Kroll LE, Muters S, Stolzenberg H: [Measurement of the socioeconomic status within the German Health Update 2009 (GEDA)]. Bundesgesundheitsblatt Gesundheitsforschung Gesundheitsschutz 2013; 56: 131-43.

e2. Robert Koch-Institut: Bundes-Gesundheitssurvey: Alkohol Konsumverhalten in Deutschland 2003. [www.rki.de/DE/Content/Gesundheitsmonitoring/Gesundheitsberichterstattung/GBEDownloadsB/alkohol.pdf?__blob=publicationFile](http://www.rki.de/DE/Content/Gesundheitsmonitoring/Gesundheitsberichterstattung/GBEDownloadsB/alkohol.pdf?__blob=publicationFile) (last accessed on 9 December 2020).

e3. Lowe B, Grafe K, Zipfel S, Witte S, Loerch B, Herzog W: Diagnosing ICD-10 depressive episodes: superior criterion validity of the Patient Health Questionnaire. Psychother Psychosom 2004; 73: 386-90.

e4. Kroenke K, Spitzer RL, Williams JB, Monahan PO, Lowe B: Anxiety disorders in primary care: prevalence, impairment, comorbidity, and detection. Ann Intern Med 2007; 146: 317-25.

e5. Michal M, Wiltink J, Kirschner Y, et al.: Complaints of sleep disturbances are

associated with cardiovascular disease: results from the Gutenberg Health Study. PLoS One 2014; 9: e104324.

e6. Campbell N, Gaston A, Gray C, Rush E, Maddison R, Prapavessis H: The Short Questionnaire to Assess Health-Enhancing (SQUASH) Physical Activity in Adolescents: A Validation Using Doubly Labeled Water. J Phys Act Health 2016; 13: 154-8.
